# Exploring how to sustain ‘place-based’ rural health academic research for informing rural health systems: a qualitative investigation

**DOI:** 10.1186/s12961-020-00608-7

**Published:** 2020-08-12

**Authors:** Belinda O’Sullivan, Alice Cairns, Tiana Gurney

**Affiliations:** 1 The University of Queensland Rural Clinical School, Locked Bag 9009, Toowoomba DC, QLD 4350 Australia; 2 Centre for Rural & Remote Health, James Cook University, PO Box 341, c/o/Weipa Hospital Lot 407, John Evans Drive, Weipa, QLD 4874 Australia

**Keywords:** Rural health research, Place-based, Capacity-building, Sustainability, Turnover

## Abstract

**Background:**

The field of rural health research is critical for informing health improvement in rural places but it involves researching in small teams and distributed sites that may have specific sustainability challenges. We aimed to evaluate this to inform how to sustain the field of rural health research.

**Methods:**

We conducted In-depth semi-structured interviews of 50-70 minutes with 17 rural early career researchers who were from different research sites across rural Australia. Data were thematically coded.

**Results:**

Seven sustainability challenges were noted, namely recognition, workload, networks, funding and strategic grants, organisational culture, job security, and career progression options. Rural researchers were poorly recognised for their work and researchers were not extended the same opportunities enjoyed by staff at main campuses. Unpredictable and high workloads stemmed from community demand and limited staff. Strategic grant opportunities failed to target the generalist, complex research in this field and the limited time researchers had for grant writing due to their demands within small academic teams. Limited collaboration with other sites increased dissatisfaction. In the face of strong commitment to rural ‘places’ and their enthusiasm for improving rural health, fixed-term contracts and limited career progression options were problematic for researchers and their families in continuing in these roles.

**Conclusion:**

A comprehensive set of strategies is needed to address the sustainability of this field, recognising its value for rural self-determination and health equity. Hubs and networks could enable more cohesively planned, collaborative research, skills sharing, senior academic supervision and career development. Targeted funding, fit to the context and purpose of this field, is urgent. Inaction may fuel regular turnover, starting after a researcher’s first years, losing rich academic theoretical and contextual knowledge that is essential to address the health of rural populations.

## Background

Around half of the global population lives rurally but rural health outcomes continue to significantly lag behind those of metropolitan areas [1]. The field of rural health research, involving trained researchers, based in rural communities and regions, working in trusted partnership with rural stakeholders to inform improvements in rural health, has an important role to play in addressing these disparities [2]. As opposed to ‘place-neutral’ evidence, rural health research is centred on ‘place-based’ spheres of influence on health outcomes, including population and culture, distance and isolation, workers and resources, health services, and practice models. For this reason, rural health research is essential for informing tailored solutions in rural settings over ‘place-neutral’ evidence that ignores the nuance of rural contexts.

The field has a strong record of producing significant evidence that has informed major rural policy reforms, service models, community action and rural health problems [3–8]. WHO urges more rural health research to inform policy strategies about various health workers and contexts and to identify not only what works, but how and why [9]. Likewise, consecutive major rural health strategies call for more rural health research to inform ongoing policy and programme priorities [7, 10, 11]. Moreover, the availability of research opportunities is integral to attracting and retaining rural health workers [12, 13]. However, understanding how to sustain rural health academic research is a significant issue given that these researchers may work in different ways to metropolitan-based academics, including working across a generalist skillset, in small teams and distributed sites, and their field is depicted by responding to high levels of community demand with limited funding [2] [14]. Australia, the United States and Canada are all countries that have formally sought to or embed rural health research capacity; however, none has evaluated the sustainability challenges of their models [14–17]. We aimed to evaluate the sustainability challenges of researchers in this field, specifically to identify how to sustain rural health research capacity.

Developing doctoral level research-trained staff in any field is a long-term investment of universities and involves supervised training for tertiary qualified candidates over a minimum of 3 full years as they navigate the research process to produce new knowledge. Atop of their doctoral training, other studies suggest that rural health researchers are required to rapidly diversify skills in the post-doctoral period in order to respond to the breadth of community demand with few other academics onsite [2, 14]. Further, the field relates to researching within trusted partnerships, applying rich theoretical and contextual knowledge of people and place that takes time to develop. This is not easily replaced by employing new staff, many unfamiliar with the rural health context and rural health topics, or who see limitations to living and working in rural places. As a result of these factors, turnover of rural health trained research staff may cause major disruptions to community engagement and workflow and impose increased workload on remaining academic staff. In turn, progress towards health outcomes in rural and remote communities may be affected, reinforcing existing health inequalities [18]. As such, a critical issue for countries wanting to build rural health research capacity is having a clear strategy for researcher sustainability.

Most of the literature about sustaining the rural workforce relates to rural clinical workers, showing that turnover and retention worsen with increasing remoteness [19, 20], related to lower-grade positions [21]. Improving sustainability is considered to rely on multilevel approaches that holistically address supportive workplaces, career development options and place-based attachments [22, 23]. This aligns with work motivation theory, where holistic factors, including the context, may be predictive of attachment to and experience of various employed roles [24]. There is no known evidence about turnover considerations of rural health researchers. Limited research about interventions to foster rural clinicians doing research identified the key sustainability challenge was having enough time to do research work [25]. Rural clinicians already face higher clinical demands and wider scope of practice than metropolitan practitioners such that research may be hard to prioritise within their portfolio [26–31]. Strategies to sustain a specialist rural health academic workforce may have other sustainability challenges but investing in trained academic researchers is important to add to the overall employment growth of rural areas, increase the level of  community access to high quality education and build capacity for rural clinicians. These factors underpin rural aspects of the Sustainable Development Goals [32]. Broader research, not specific to rural areas, suggests that deliberate interventions to develop early career researchers (ECRs) capable of working in multi-disciplinary teams and solving complex real-world problems is important for retaining talent [33]. Understanding sustainability in the rural health research context is important given that both researcher career stage and discipline relate to research development outcomes [34].

### Context

Our research was based in Australia, which is an isolated continent nation, with coastal cities, (small by global standards) of at least a day’s car travel from each other. These cities are positioned within wide expanses of coastal and inland rural and remote towns of distributed populations and arid unpopulated areas. Different levels of hospitals and community health services, including Aboriginal Community Controlled Health Services, private GPs and government health agencies, operate in rural and remote towns across Australia. Over a 20-year period, Australia has expanded policies and training programmes to improve rural health [35, 36]. One of these policies was an Australian government policy called the Rural Health Multidisciplinary Training (RHMT) programme, which commenced in 1996 to fund universities to develop rural infrastructure for training health workers and, alongside of it, employ rural health researchers that typically work on 3-year contracts [16]. The government contracts expect these researchers to cover issues of training, rural workforce, models of care, rural health topics and Indigenous health. The research outputs from the RHMT programme have already been described in the literature [37, 38]; however, the sustainability challenges for rural health researchers have not. A government review of the RHMT programme was commissioned in 2019, and we considered it timely to explore this topic and to inform ongoing approaches to foster this research field in Australia whilst also providing information that may support rural health research systems in other countries [39].

## Methods

In-depth semi-structured interviews were conducted with 17 rural ECRs (<8 years from full time equivalent from doctoral thesis award), residing in Australian regional, rural and remote locations. ECRs were selected as they constitute the bulk of rural research academic staff and the ECR period is identified as requiring specific support and planning for sustainability [40].

### Procedure and semi-structured interviews

Subjects were recruited via Australia’s Federation of Rural Academic Medical Educators (FRAME) and the Australian Rural Health Education Network (ARHEN). On our behalf, they sent information about the study to the representatives in constituent organisations of 16 University Departments of Rural Health and 20 Rural Clinical Schools, with an enclosed package that they could forward to invite ECRs to participate. Snowballing via email was encouraged to reach other rural researchers external to this network. No reminders were given as the study was well subscribed. Purposive methods sought to include researchers with different characteristics such as sex, age group, rurality, distance from main university campus and rural origin (Table 1), remoteness of locations and experiences [41]. Eligibility required researchers to live and work in areas Modified Monash Model (MMM) 2–7 (rural areas by Australia’s definition) [42] and be working actively in rural health research, defined as workforce, models of care, rural or remote population health, rural health services or Indigenous health for >50% of their active research time.

**Table 1:** Characteristics of rural health researchers (*n* = 17)

Factor	n (%)
Sex	
Female	13 (76)
Male	4 (24)
Age group	
30–39	8 (47)
40–49	7 (41)
50–55	2 (12)
Rurality of research location (MMM)^a^	
2	5 (29)
3–5	9 (53)
6–7	3 (18)
Distance from main campus	
<200 km	5 (29)
200–399	6 (35)
400+	6 (35)
Primary income earner	
Yes	10 (59)
Trajectory to this position	
PhD qualified then moved to rural area with partner’s work and found this job	2 (12)
PhD qualified then moved to rural area to take up this job	8 (47)
Already living in rural area, got PhD qualified and found this job	7 (41)
Childhood origin in rural location of current work	
Yes	3 (18)
No	14 (82)
PhD was in rural health	
Yes	5 (29)
Employed in a government contracted position	
Yes^b^	14 (82)
No	3 (18)

^a^Modified Monash Model (MMM) is the Australian government’s geographical scale that denotes areas that are metropolitan, rural, remote or very remote, based on population size and remoteness. MMM2–7 defined rural areas (https://www.health.gov.au/health-workforce/health-workforce-classifications/modified-monash-model)

^b^82% of the rural health researchers were employed on government contracts under the Rural Health Multidisciplinary Training Fund which at the time of this research had 15 months remaining, with the potential for renewal pending a government review which commenced in 2019

After completing informed consent, a recorded videoconference interview of 50–70 min duration occurred between August and December 2019. Each interview was transcribed verbatim. Data collection continued until authors agreed to thematic saturation. During the interviews, participants were asked to describe their research background, training and rural health research career experiences, specifically reflecting on sustainability issues within their current role. Prompts were used to expand on emerging themes (see Table 2 for the interview schedule).

**Table 2:** The interview schedule

Focus area	Questions
About you	Age, sex, how came to live and work where you do? Rural background
	What is your background (skills, quals and why you did a PhD)?
	What was your PhD about and when did you complete it?
	What are your main research skills (quant, qual etc.)?
	How did you come to work in rural health? (motivations)
	What is your current main research focus with respect to “rural health” (people, places, workforce, services, outcomes, Indigenous etc.)
	If your main research focus is different to your PhD research how/why did this come about?
	Where is your total full-time equivalent (FTE) currently divided now (research FTE, other FTE, interests)?
	What are the most enjoyable things about researching rural health?
	What are the hardest things about it?
	What other responsibilities do you have in your community, whether that be for family, community, other roles?
	What are the practical challenges you face, if any, as a rural or remote person?
The unit	Tell me about the ‘rural health’ research culture in your unit (who employs you, how much active research is happening around you, do you have a research plan)?
	How long have you worked here?
	What are the main stakeholders you work with, where?
	To what extent is your work meaningful and why?
Research careers	Now I am going to talk about some factors common to research careers and ask you to reflect on some of the challenges or enablers you experience with these in the rural setting and why?
	Security of employment?
	Access to supervision of a more senior academic?
	Sustainable workload?
	Attracting small pots of grant money to tie you over?
	Attracting larger grants?
	Attracting PhD students?
	Publishing, in high quality (Q1) or international journals?
	Writing protocols and ethics?
	Conference attendance?
	Research translation?
	Research infrastructure (data available, internet, library support, ethics online, human resources systems)?
	Research support staff?
	Connections with health services, organisations?
	Collaborating with others?
	Demonstrating leadership in your field?
	Of these things, what are the main factors affecting your research career progression right now?
	What would most help you and why?
Intention to stay	How long do you think you will be able to sustain yourself working in this research role in this location?
	Are you currently or considering looking around for a new job? If so, in what sort of field and why?
	What would you miss about rural health research if you were not doing it?
	What would others miss if you did not do the role you did and it was not back-filled?
	Overall, how would you describe your overall job satisfaction as a researcher in rural health?
	Where do you want to be in the next 5 years?

Ethics approval was obtained from Monash University Ref. no. 20595, ratified at University of Queensland and James Cook University.

### Data analysis

To build understanding of the data, all three authors conducted at least three interviews, recorded notes post-interviews and met regularly to discuss emerging themes. Once interviews were completed, theoretical and inductive thematic analyses were undertaken. Theoretically informed analysis involved the authors (all rural health ECRs) applying their knowledge of the broader literature about rural systems and retention [43–49]. Further, the theoretical approach for this study was informed by work motivation theory suggesting that the ability to predict, understand and motivate people requires holistic consideration, including of work context – where national culture, characteristics of the job itself and the fit between the person and the organisation may influence motivation [24]. Additions and alterations to the coding framework were made as blocks of five transcripts were completed, shared equally between the authors. Authors then double coded another transcript identifying reasonable concurrence with existing themes but adding extra codes where these applied. Inductive coding specifically built on these codes, whereby data elements were considered without using a pre-existing framework, so that the themes were further developed, strongly related to the data [50]. This involved in-depth review and analysis led by the main author. The findings were discussed by the team, checking the data for internal corroboration of disconfirmation, reaching consensus on the final themes as a research team [43, 51]. In the process, the team aimed to promote self-reflexive analysis by considering the influence of their own experiences on the interpretation of data, by extensive written and verbal reflection by team members [52, 53]. To ensure separation of the findings from our own roles as rural health researchers, thereby minimising bias [54], interpretations of the data were tested by regular group discussion and each team member only interviewed participants not working in the same research unit. The process of analysis involved multi-layering over a 6-month period, until thick description and triangulation of findings occurred for confirmability [55].

To aid interpretation of the data, responses were reported as relating to the number of participants: 1–4 participants as ‘*few’*; 5–8 as ‘*some’*; 9–13 as ‘*many’* and; 14–17 as ‘*most’*. Further interviews were classified according to whether the ECR was based in a regional (MMM2), rural (MMM3–5) or remote setting (MMM6–7) using ‘reg’, ‘rur’ and ‘rem’, respectively, as subtext for the quoted material [42].

## Results

The research participants are described in Table 1 and were from 6 of 8 Australian states/territories, including Tasmania, Victoria, New South Wales, Queensland, Northern Territory and South Australia, with a wide representation of most (71%) working in smaller towns than major regional centres (MMM2 which is >50,000 population) inclusive of 18% from very remote locations (MMM6–7). Seven key sustainability themes emerged (Table 3), namely recognition, workload, networks, funding and strategic grants, organisational culture, job security and career progression options. Further, tipping points were noted as a way of contextualising the dynamics of particular factors with respect to leaving the field*.*

**Table 3:** Themes related to sustaining rural health research^a^

Theme	Description
Recognition	Poor recognition by main university campuses and their own units for effort and achievements and the value provided to community
Workload	Small academic teams researching large problems, high demand to take all opportunities and respond to the community interest in researching and solving rural health issues
Networks	Working solo, geographically isolated, with few opportunities to connect to rural health researchers in other sites and limited senior academic mentoring from others in the rural health research field
Funding and strategic grants	Structural barriers to writing grants (nature of field complex makes it hard to confine to a grant topic/simple method, few team members and time for research outputs and grant writing), limited grants target improving health in rural-places
Organisational culture and leadership	Poor leadership and encouragement of collaboration with other sites affecting cohesive business planning and job satisfaction
Job security	Ongoing job security insufficient relative to the commitment researchers made to rural places
Career progression options	Employed and maintained at levels below competence and experience

^a^The themes are overlapping and reinforcing, rather than linear and causal of poor sustainability on their own

### Recognition

Issues of recognition operated on multiple levels. One noted that there was increasing focus on urbanity, as a competitor to rural ‘place-based’ research:

“*… there’s lots of conferences about urbanity at the moment … that kind of privileging of metro*[politan] *and urban and you’re trying to carve out a space and say rural is important … predominantly to decision makers who are probably sitting in cities*.” (Reg 5)

Rural health research work was poorly recognised at one researcher’s main university campus as it did not often fit the boundaries of clinical trials, within a biomedical model:

“*I’m embedded in the medical school so none of my research ever gets valued because it’s not clinical trials*.” (Reg 6)

Many noted that it was merely a factor of out of sight out of mind, in terms of respecting the research done, its context and the opportunities granted to researchers at distributed sites (away from main campuses):

“*… we are west of the* [X]. *You sit there and go, you forget that we exist and we’re just out here by ourselves.*” (Rur 8)

“*… the* [main campus] *researchers without a doubt are offered opportunities over anyone else simply because they’re in close proximity.*” (Rem 1)

Some noted that their career achievements in rural health research were also poorly recognised in their own unit where the focus was on student clinical education:

“*… my* [X] *Fellowship was celebrated, actually, even at the top university level, but not in my own department. The achievements that I’ve made in the main are my own. Through my own hard work and almost in spite of the lack of support and recognition …*” (Rur 1)

“*… the approach is very much bums on seats, all teaching, and there’s very limited infrastructure, very limited - you know anything you do, like any academic I guess in* [X]*, you do everything out of hours. It’s all just out of the good of your heart.*” (Reg 6)

Finally, some also mentioned that they were not valued for their own research, but only as a service arm or relegated as a data collector for metropolitan researchers:

“*… you are not just a data collector for a big team sitting in* [X] *or* [X]*. We are equal partners...*” (Rem 3)

“*I know there are a lot of researchers who work in my region who come from* [X]*. They have big clinical trials that are running here, or they are going into communities and delivering interventions … They just come into your community and do their business and go...*” (Reg 1)

### Workload

For many, the feasibility and unpredictability of the academic workload was an issue due to the small team size, the community demand, researcher enthusiasm for meaningful projects and the imperative to accept new research opportunities:

“*I think when you are in an environment where there are a limited number of people who have a certain capacity and you happen to be one of those people I think the workload can get quite out of control quite easily...*” (Rur 4)

“*… you can’t say no to projects, Because you don’t often get those opportunities. In saying yes to a project on top of your current load, nothing’s ever taken away from you, your workloads never reduce...*” (Rem 10)

“*The way it generally works is the clinicians want to do a research project. They come to me and ask me if I’d be interested in helping them, and that usually turns into me taking the lead on the research.*” (Rur 2)

Some expressed having too much work relative to their employed fraction in research and pursued research in unpaid time:

“*I do it between eight and ten at night. If I do it at all. If I’m not exhausted, I have* [X] *kids and a husband and a dog and three chickens. You sit there and you code data in your own time. That’s the only way it gets done.*” (Rur 8)

In part the high workload was exacerbated by small team size and also by the breadth of skills needed in the face of more professional isolation:

“*If I’m stuck* [with some] *problem … if I were part of a bigger team there would be many statisticians sitting in the* [far] *group and they would be happy to help … Here, sitting alone, you need to figure it out by yourself … I have to go through hours and hours of Google -* [surfing] *and looking for resources.*” (Rem 3)

“*It probably takes me 10 times as long to do things that people in* [X] *are doing like that, because I don’t know the faster way to do it or the better way to do it.*” (Rem 1)

To balance the workload researchers learnt to respond by prioritising the large number of opportunities around their skills:

“*There’s obviously lots of work to be done. I think that’s a good and a bad thing. It means there’s lots of potential opportunities. Being able to select ones that suit you better is a challenge, in terms of the skill set that you might have....*” (Rur 7)

“*You’ve got to learn to say no because there is so much you can do and get involved with.*” (Rem 5)

### Networks

Networks with other rural health researchers were considered important over collaborating with metropolitan researchers, as the former encompassed academics with knowledge of the rural context, considered an enabler of meaningful exchange:

“*So I’ve very much been a solo researcher working on this … it’s very place based research. I’ve got more in common with those researchers who are outside my university than the ones in* [city] *University.*” (Rur 1)

“*So actually in terms of being connected I feel better connected to my rural colleagues than I do to any metropolitan area*” (Reg 6)

However, few had access to rural research networks and if they did, these were localised:

“*I’m part of the* [X] *Research Network, I don’t know if you’ve heard of them. It was something that was started by* [X] *… that’s been a big deal for me … a very supportive environment to be involved in...*” (Rur 7)

Some addressed networking by developing their own connections but finding the right people with whom you ‘belonged’ was challenging due to the distributed structure of the field:

“*I have pretty emerging relationships with researchers and I’m kind of shopping for them as I need them and then building my own team.*” *(Rur 1)*

“*… we’re really siloed … how do we find that person who’s willing to take the time to invest in you if I can’t sit there and knock on their office door and say, ‘hey can I get you coffee?’*” (Rur 8)

“*I think that’s always a struggle with that multi-site stuff, feeling part of … your colleagues and things like that …*” (Rur 3)

Within professional networks, the need for senior rural health researchers was noted to enable more opportunities:

“*… there is also a lack of that senior, like academics mentoring.*” (Rur 5)

“[A] *strong network of other more senior rural researchers who have a common thread …, they need to be collaborative in creating opportunities for funding and bigger research projects.*” (Reg 6)

Many felt that senior academic mentoring independent of the line manager, was critical for intellectual extension:

“*… I crave mentoring … I don’t have research supervision. I have line management. I’m hungry for … other researchers critique … I could just be more confident if I had senior researchers. I don’t.*” (Rur 1)

### Funding and strategic grants

Many researchers raised that funding and strategic grants were not easy to access due to the lack of funding streams fit to their field:

“*I actually think that the funding streams probably don’t fit well with the rural health issues … the types of methodologies and things that bode well in this area, they’re just not sexy enough for competitive funding.*” (Reg 6)

“*… the research you’re doing connected to place, as a place based researcher, is extremely complex … the complexity of individual health services, communities, pathways are dislocated and it is extremely complicated to track people.*” (Reg 2)

“*Even though there’s this massive area of need and all the governments are saying this is an area of need … the grants aren’t really quite aligning with that, or where they’re going isn’t aligning with that need.*” (Rur 3)

Some researchers also experienced structural barriers to grant writing either directly from the time the unit gave them:

“*… the priority is teaching students, supporting students, and also having the capacity to look up and create those networks and to put the effort into a big grant is just unrealistic.*” (Reg 6)

The size of the team:

“*Being part of a bigger team where I can get some grants and do some work in the area* [I want to do].” (Rem 3)

Or the challenge of building sufficient track record:

“*… you’ve got at a time maybe six, seven publications at various stages and you’re just trying to get them done and get them published so that you can show you’ve been actually doing some work in the past four years.*” (Reg 3)

### Organisational culture and leadership

The characteristics of rural research unit leadership and management affected satisfaction with work. Strong leaders who could work with diverse and distributed teams and who understood rural research were valued:

“*… the boss we had … she was really great. She had this vision. Okay, I’ve got this team and … how could I put them together?*” (Rur 3)

Others reported a lack of cohesive business planning related to distributed sites and managers with limited research skills:

“*… We get together and we have meetings regularly so people can talk about what they’re doing …* [there’s] n*o research plan in place, no clear career goals of working towards in research, it seems to be very ad hoc*.” (Rem 1)

*The research leader, they don’t have a vision or plan. Some days they will come up with some random idea and then it will be super busy …*” (Rem 3)

Access to direct research leadership and clear research focus were enhanced when researchers were employed on existing competitive grants that had senior research leaders:

“*I have other more senior academics who are involved with our projects who do provide a lot of support and guidance to me … So, with my supervisors right now I have it whenever I need it …*” (Reg 3)

Or, for some, when the research leader was on site:

“*… So, this is the only site which is very much active in research, because the research lead's here …*” (Rur 6)

“*… you have ideas and they'd say go for it … There's a real culture of mentoring and building capacity*.” (Reg 2)

Some government contracts were affected by the focus of maintaining government funding and building university reputation, rather than fostering the development of local staff and building multi-university research collaborations:

“*That's sort of part of the mentality of reporting to government to say we're doing everything, we're functioning well, yeah. But there's a lot below that in terms of the experience for the people in units*.” (Rur 4)

“*I didn't feel … that we were encouraged to actually create those collaborations outside of our own little bubble ...*” (Rur 4)

### Job security

Relative to the commitment to place that doctoral-trained research staff made to participate in rural health research, ongoing job security in relation to 3-year rolling government contracts was an issue for job satisfaction:

“*… the hardest thing is it's contract work, so you don't know where … you're going to be after that. So there's a lot of instability...*” (Rur 3)

“*I think we have a solid research culture here. But it’s only for the last two and a half years. All this is threatened by who is not going to have contract tomorrow or there’s no funding …*” (Rur 5)

These contracts also impacted the capacity to attract students:

“… *because of our funding cycle we actually try to get a PhD student in on scholarship, but we have a two-year funding cycle and a PhD takes three years*.” (Rem 4)

For one researcher who was the primary income earner, the inability to gain tenure was also a deterrent:

“*I mean one of the major limitations for me was there was no possibility that I could ever be continuing in that position … I'm like the sole income earner for my family, my partner is a stay at home parent, so that was really significant for me...*” (Rur 4)

Others, who had experience in working with government contract periods, were not concerned about job security.

“*We operate on two- and three-year contracts, but I don't let that bother me, because in the 16 years I've worked here my job - the contracts have continued …*” (Reg 1)

Others employed in rural areas on competitively funded grants noted specific funding challenges:

“*As long as there's funding I think it's sustainable. But again, it's a real gamble...*” (Reg 3)

The broader job opportunities in the community, in the event that research contracts were not renewed, were also a strong consideration for many researchers and their families:

“*Probably the challenges specifically related to* [X] *are probably just job opportunities …* [for] *myself and my partner*.” (Rur 3)

“*… when you're coming up to the end of your contract, you need to be actively looking because there may be no other job for another year. There's not that many employers. So, what do you do? Where do you go?*” (Reg 5)

### Career progression options

Many rural health researchers commented that they were employed and maintained at levels below their competence and experience. Others had particular achievements, like grant funding to address to advance their career, which was difficult being based in a rural area:

“… *when I look at the level As, there are research assistants working to people's agendas. I'm working to my own agenda *… [with] *international partnerships established. So how is that a level A? Actually, it's closer to a level C than it is to a level A.*” (Rur 1)

“*I suppose the other one is that I've been at level A the whole time that - I was originally contracted as a level A and I am still a level A....*” (Rur 2)

“*I've been a Level B forever in a day and I can't see myself moving to the next level because … I need a Category 1 grant and that's just not going to happen*.” (Reg 6)

The generalist nature of their research role made it challenging to demonstrate academic achievement on mainstream research metrics:

“*… you create your academic career on becoming more and more and more and more specialised and that doesn't bode well for rural health*.” (Reg 6)

Some also mentioned that they particularly needed strategising about how to navigate a research career:

“*It would be really good to have someone supporting you on how you might navigate that* [research career]*. Or twist things, ‘going do you realise that that is that? Or you should be promoting that more as something that you do on the side’*.” (Rur 1)

Others noted that there was not a visible career path in rural health research:

“*I think it's probably about having that mid, maybe mid-career researchers. We've got early career, we've got, there's more senior people, but let's look at that mid-body*.” (Rur 3)

For others, they were hopeful that career progression options would follow in time:

“*… it’s just a Research Assistant’...at this stage, I am mentally prepared to stay here at least for one year. I will see* [what] *would be the other opportunities, but my team leader has said, currently this is the only position we have …*” (Rur 6)

### Tipping points

The intention to stay and satisfaction with various conditions varied over time for some rural researchers. Some were happy to stay in rural health research in rural places until retirement:

“*I could probably stay here until I retire I think*.” (Rur 2)

Some were happy with the role, but these were mainly in their first 6 months of the position:

“*… for me at the moment, it just feels like an absolute privilege to just be able to do research and get paid for it*.” (Reg 5)

“*… I love those light-bulb moments; I love to read the literature and find out what's going on. I do love knowledge and I think this job has that for me*.” (Rem 5)

Those in their second and subsequent years of the same position more so reflected on being in a state of tension over whether to stay or leave because they viewed their work as valuable to rural communities and their work had been built up from substantial concurrent investment. One who was 2 years into a part-time research position mentioned:

“*… I’m struggling, probably with my situation at the moment is because it’s such important work … even for all the flaws of our research team … this is such important work.*” (Rur 8)

“*I think it's a terrible shame because what that seven years of investing in this research, that's quite a bit of investment from government and I walk away …*” (Rur 1)

Leaving was particularly disappointing for ECRs who moved their families to rural areas to pursue their research in the field:

“*Yeah, I think I was just gradually disappointed. Six months in, I was concerned. But you know, I'd moved myself and my husband here. We'd disturbed our entire life. … I've been working pretty hard to make sure that the whole thing wasn't a dreadful mistake*.” (Rur 1)

Of our sample of 17, 3 researchers had already resigned to work in different academic/policy fields and 2 were contemplating on leaving. Of those who had resigned from working in rural health research roles, the main tipping point was employment arrangements and poor research leadership culture:

“*… I love the place, I love my team, that leadership has been really disappointing …*” (Rem 3)

“*I love being a researcher... Yeah, it sits around nine out of 10 … there’s many things I can do. But the employment arrangements are about a three … So then you come back to about a five or a six*.” (Rur 1)

“*I've resigned from my position, and the culture is one of the reasons why I resigned … . It's been just lack of support really …*” (Rur 7)

Another researcher noted the inability to achieve a successful research carer in rural health but signed another contract to continue working in the location as she had moved her family to the area so felt obliged to stay in the role:

“*… In my mind I’m done, I’m not building a research career, the clock’s ticking... I only signed another contract because I had my family out here and they didn’t want to go here, but I’m done. I can’t see the support that I’m receiving's going to change.*” (Rem 1)

## Discussion

This is the first national-scale study to evaluate the sustainability challenges of rural health researchers in distributed sites, most of whom were are least 200 km from their main university campus and mainly (82%) working in government-contracted research positions. It identifies seven clear challenges that inform a framework for countries considering building sustainable rural health research capacity. This work builds on other rural workforce retention frameworks that have been limited to clinical workers [9, 12, 22]. It also aligns with motivational theory, reinforcing that holistic approaches are needed to sustain work, including attending to context as a driver [24].

Most participants identified that their research work was not recognised or valued and they were not extended research opportunities or resources from their main university campuses. They were “*just out here by ourselves*”, with “*limited infrastructure*”, although competing within a mainstream culture privileging controlled and scaled-up research such as clinical trials. This is likely to be a product of strong research market competition for both jobs and grants in Australia. However, improving the recognition of rural health researchers and their research is a critical societal issue central to overcoming geographical narcissism, where cities ‘other’ and ostracize rural places and their issues and research as ‘less than’ and hold resources for themselves [56]. Such an approach significantly disadvantages the production of research that fits rural settings as well as the community partnerships needed for rural system translation of findings. Using rural researchers as data collectors for city projects or bypassing them altogether by ‘fly-in, fly-out’ projects is symptomatic of a research culture where skills and resources are owned by cities, and the genuine skills and efforts of trained rural health researchers are devalued [2, 14].

Within their own rural units, our participants also faced lack of recognition partly because the ‘*priority is teaching students*’. Under-resourced settings may preference pragmatic service-orientated fields over conceptual ones, like research. Yet, just as rural services capacity needs to be built, equity of access to academic skills in rural areas is also inherent to lifting rural societal opportunities [32]. Perhaps better espousing the value of rural research as part of the overall agenda for informing broad-scale strategies and community action towards rural health equity may assist in re-framing any perceptions that research is a mere luxury that only cities should be permitted to access [36, 56]. Our findings show that rural health researchers are sustained by internal reward systems – “*none of my research ever gets valued*”. This failure to give due recognition for rural health research work is likely to lead to professional dissatisfaction and reinforce professional isolation, as key factors related to turnover [49]. Other literature points to the value of using professional rewards to reinforce the values and norms of people’s roles and thereby promote the achievement of organisational objectives, in this case, of contributing to solutions for rural populations and health services [57]. Some options include universities and their rural sites introducing academic awards and simply noting the contribution that rural researchers are making to their communities, as a relatively cost-neutral strategy.

Due to the drive to be accountable to communities and our participants’ enthusiasm and culture of opportunism, our participants had unpredictable and heavy workloads – “*you do everything out of hours*”. This may be worsened by the finding of poor leadership impacting uneven and limited cohesiveness of research business planning coupled with siloed decision-making. To some extent, a high workload was normalised by a rural culture where overwork is reinforced as a demonstration of dedication [58]. However, compared with rural clinicians, rural health academics had limited staff and skill-shifting options to buffer workload, including few other trained staff for the breadth of skills needed. The scenario of being “*stuck* [with some] *problem*” involving “*hours and hours of … looking for resources*” highlighted challenges that could result in turnover. More sustainable than relying on individuals to remain reslient in the face of this pressure, is to use a systems-wide approach. For example, initiating more rural research hubs (virtual or physical) at regional, state or national levels, as a model used for rural research in the United States [15], could assist researchers at individual sites to access a suite of skills and senior academic supervision for trouble-shooting. This is likely to be more cost-effective than building the numbers of research employees at each site and it may also address the identified need of rural health researchers “*crave*[ing] *mentoring*”, “*critique*” and “*senior researchers*” familiar with their context, as the basis of meaningful exchange. However, the risk is that hubs unwittingly draw resources away from sites, which could be attended to by having a clear mission, principles and service contracts.

Other countries have used nationalised rural health research networks to foster rural health research capacity [7, 17]. Our participants were already seeking their own networks or joining regionalised networks but access to these was inequitable depending on the researcher's region. Additionally, our broader findings suggest that a network alone may be insufficient to improve sustainability in this field. This is not to say that if they were integrated within a wider range of interventions and connected with hub nodes, networks may support rural health researchers to move “*outside of our own little bubble*” and become part of a dynamic community of practice, where people mutually guide each other through their understandings of the same problems in their area of passion. Indirectly sharing tacit knowledge through regular exchange (beyond any one national conference, presentation or review of circulated material) is expected to engage people in the collaborative learning process of ‘thinking together’, which is central for communities of practice to come to life [59]. This contrasts with our participants’ experience of discussions at sites being limited to local and pragmatic problems like government reporting. Instead, a community of practice may provide for the intellectual extension that was craved.

In turn, addressing sustainability by using hubs and networks could also enable more collaborative and strategic grant funding opportunities to flow to small sites and support the capacity to access PhD students, achieve research outputs and provide career development opportunities. Participants in our study noted that there was limited funding or strategic grant opportunities targeting rural health research and a competitive culture between units as a potential limitation for cross-site collaboration. They felt their generalist field of co-designed research and complex issues and methods done with few academic staff, over a distance, did not fit the tight criteria of grant funding and made it challenging to address the level of outputs required to be competitive as well as the time to write grants – “*the grants aren’t really quite aligning*”. A sense of empty rhetoric about the grant funding commitment for rural health was noted given the limited availability of other funding streams and the fit to the field’s characteristics. Our researchers’ perceptions reflect the findings of a review of National Health and Medical Research Council grant funding, which confirmed only 1.1% of research funding reaches rural health (2000–2014) despite 29% of Australia’s population living rurally [60]. Similarly, another competitive funding mechanism, the Medical Research Future Fund (expected to grow to $20 billion in 2021, based on the 2018–2021 plan) does not include rural health as a specific topic area [61]. It is urgent that major funding streams intended to address the health of all Australians, better targets this field and accommodates its nuance [2]. Given that rural health research supports social and economic growth, rural education and attracts rural clincians, each being major issues for the 'rural' public good, capacity still requires core government funding to supplement geographically targeted competitive grants [2].

Despite the strong commitment that our participants (and their wider families) made to rural ‘places’, time-limited employment contracts affected researchers’ security, family location decisions and the strategic growth of their research. The lack of tenured positions mainly impacted primary income earners for whom the need of career stability was a central issue. This could largely be mitigated by providing a mix of tenured options and longer-term funding that foster ongoing engagement and partnership work in rural health research. Governments may need to consider this given that competitive grant funding is often short-term and currently hard to access in rural areas [60]. Further, most of our participants reported limited career progression options in their field and frustration about the metrics needed to achieve career progression. This is highly likely to be affected by poor career mentoring for academic career navigation, one participant mentioning their academic career “*clock’s ticking*”. Career support, security of employment and mentoring are also issues for mainstream researchers. However, academic career progression may be poorly respected within rural settings where people have fewer alternative job options and have committed themselves and their families to a place. Compared with urban research, by virtue of working in underserved environments, collective gain may be a core focus of the researchers work; however, for sustainability and career progression, it is urgent that rural health researchers also have the right levels of be support to achieve personal gains like career milestones. This capitalises on their investment in a research career and assists them to maintain career mobility within a rigidly structured profession that currently does not have rural-specific weightings. Other research shows that ECRs, as a broad group, may stay in existing positions longer than planned due to limited salary, training and job opportunities [62]. Strategies to sustain rural health research must include tailored career development discussions with more senior mentors (virtual or in person) in their field.

Our research showed that career dissatisfaction increased quickly, after the first year, mainly because of factors related to the employment and organisational culture. The decision to leave rural research work involved a huge tension about forgoing love of the field and the investment in their research and academic skills. The joy of rural health research has been described in other research, including allowing researchers to experience the whole spectrum of research, covering diverse topics, enabling researchers to stretch, grow and connect to people and places [2]. Leaving was viewed as having the potential to affect the community “*it’s such important work*” as well as the researcher’s own investment in growing their research programme, which was cause for some to ‘sit on the fence’ rather than leave in the face of the sustainability challenges they faced. Ideally, with comprehensive strategies, more would stay and could continue to build a rural health academic culture.

This study was limited to 17 researchers and, although saturation was achieved, it is possible that other themes could arise if we did more interviews. Anecdotally, most early career rural health academics are females; however, the rural health academic workforce has never been formally characterised to determine the representativeness of our sample. The study was limited to Australia, which has specific rural health research model (government funding for distributed sites). However, some of the findings may be translatable to other countries where, despite different funding methods, rural academic researchers work in isolation from each other [63]. Further research could expand or internationalise this exploratory study and consolidate our findings, including whether these issues are ongoing and how to address sustainability of this field, not just for ECRs but for rural health researchers at other stages. Although seven sustainability challenges were identified, the thematic inter-relationships could be further explored in ongoing research. There is a high probability that the themes are overlapping and reinforcing, rather than linear and affecting poor sustainability on their own. We did not identify specific themes related to being more remote although this could be explored further in a larger sample size. Additionally, this study specifically aimed to identify sustainability challenges rather than overtly seeking to understand what contributed to researchers maintaining rural health research roles. The ‘tipping points’ theme highlighted key drawcards were doing research that affected local change and being attached to people and place. It is possible that these ‘pull’ factors could be strong enough for rural health researchers to continue in their role.

## Conclusion

This is the first national cross-sectional qualitative study exploring the sustainability challenges related to the field of rural health research. Rural research work was poorly recognised and researchers were not extended the same opportunities of staff at main campuses. Unpredictable and high workloads were common due the need to respond to community demand with limited staff. Funding and grants failed to target the generalist, complex and multi-faceted nature of research in this field, nor the fact that its rural researchers had limited time due to extra travel and partnership work within small academic teams. The lack of collaborative research opportunities with other sites increased dissatisfaction. In the face of strong commitment to rural ‘places’ and an enthusiasm for their field, 3-year contracts and poor career progression opportunities were problematic for researchers, their families and developing strategic research. The current study could be extended by further surveys or tracking of rural health researchers to determine how widespread these experiences are and their exact impact on turnover. As a starting point, our study points to the need for a comprehensive set of strategies to address the sustainability of this field, mainly because it is perceived to play a strong role of informing knowledge that applies to rural settings. Hubs and networks could enable more cohesively planned, collaborative research, skills sharing, senior academic supervision and increased career development opportunities. Investing in a balance of government and targeted grants, fit to the context and purpose of this field, is urgently needed. Inaction has the potential to fuel regular turnover, starting after a researcher’s first years, losing rich academic theoretical and contextual knowledge that is essential to address health inequities in rural populations.

## Data Availability

Data and materials are protected by ethics but may be made available in de-identified format upon contact made to the corresponding author.

## Abbreviations

ECR: Early career researchers
MMM: Modified Monash Model
RHMT: Rural Health Multidisciplinary Training

## References

[1] International Labour Organization (2015). Global evidence on inequities in rural health protection: new data on rural deficits in health coverage for 174 countries, ESS document no. 47.

[2] O'Sullivan B, Cairns A, Gurney T. Understanding the field of rural health academic research: a national qualitative interview-based study. Rural Remote Health. 2020. 10.22605/RRH6116. 10.22605/RRH6116 32878447

[3] Humphreys JS, Wakerman J (2018). Learning from history: how research evidence can inform policies to improve rural and remote medical workforce distribution. Aust J Rural Health.

[4] O'Sullivan B, Stoelwinder J, McGrail M (2015). The stability of rural outreach services: a national longitudinal study of specialist doctors. Med J Aust.

[5] Tideman P, Tirimacco R, Senior D, Setchell J, Huynh L, Tavella R (2014). Impact of a regionalised clinical cardiac support network on mortality among rural patients with myocardial infarction. Med J Aust.

[6] Kildea SKS, Barclay L, Tracy S (2010). ‘Closing the gap’: how maternity services can contribute to reducing poor maternal infant health outcomes for Aboriginal and Torres Strait Islander women. Rural Remote Health.

[7] College of Family Physicians of Canada (2017). Final report: summit to improve healthcare access and equity for rural communities in Canada. The rural road map for action.

[8] Namazzi G, Waiswa P, Nakakeeto M, Nakibuuka VK, Namutamba S, Najjemba M (2015). Strengthening health facilities for maternal and newborn care: experiences from rural eastern Uganda. Glob Health Action.

[9] World Health Organization (2010). Increasing access to health workers in remote and rural areas through improved retention.

[10] Mason J (2013). Review of Australian government health workforce programs.

[11] National Rural Health Commissioners Office (2018). National Rural Generalist Taskforce advice on the development of the National Rural Generalist Pathway.

[12] Northern Periphery and Arctic Programme (2019). A framework for remote rural workforce stability: making it work.

[13] McGrail MR, O'Sullivan BG, Bendotti HR, Kondalsamy-Chennakesavan S. Importance of publishing research varies by doctors’ career stage, specialty and location of work. Postgrad Med J. 2019;95:198–204. 10.1136/postgradmedj-2019-136473 30926718

[14] Pong RW, Atkinson AM, Irvine A, MacLeod M, Minore B, Pegoraro A, et al. Rural health research in the Canadian Institutes of Health Research: a position paper prepared for the Canadian Health Services Research Foundation and Social Sciences and Humanities Research Council. Northern British Columbia: Laurentian University and Lakehead University; 1999.

[15] University of North Dakota (2000). Rural health research gateway.

[16] Australian Government Department of Health (2019). Rural health multidisciplinary training program.

[17] MacLeod MLP, Dosman JA, Kulig JC, Medves JM (2007). The development of the Canadian Rural Health Research Society; creating capacity through connection. Rural Remote Health.

[18] Australian Institute of Health and Welfare (2018). Rural and remote health.

[19] McGrail MR, Humphreys JS (2015). Geographical mobility of general practitioners in rural Australia. Med J Aust.

[20] Russell D, Humphreys JS, McGrail M, Cameron WI, Williams PJ. The value of survival analyses for evidence-based rural medical workforce planning. Hum Resour Health. 2013;11:65. 10.1186/1478-4491-11-65 PMC4029435 24330603

[21] Chisholm M, Russell D, Humphreys J (2011). Measuring rural allied health workforce turnover and retention: what are the patterns, determinants and costs?. Austr J Rural Health.

[22] Cosgrave C (2020). The whole-of-person retention improvement framework: a guide for addressing health workforce challenges in the rural context. Int J Environ Res Public Health.

[23] Russell D, McGrail M, Humphreys J (2017). Determinants of rural Australian primary health care worker retention: a synthesis of key evidence and implications for policymaking. Aust J Rural Health.

[24] Latham GP, Pinder CC (2005). Work motivation theory and research at the dawn of the twenty-first century. Annu Rev Psychol.

[25] Webster E, Thomas M, Ong N, Cutler L (2011). Rural Research Capacity Building Program: capacity building outcomes. Aust J Prim Health.

[26] O'Sullivan B, McGrail M, Russell D (2017). Rural specialists: the nature of their work and professional satisfaction by geographic location of work. Aust J Rural Health.

[27] McGrail MR, Humphreys JS, Joyce CM, Scott A, Kalb G (2012). How do rural GPs’ workloads and work activities differ with community size compared with metropolitan practice?. Aust J Prim Health.

[28] Adams J, de Luca K, Swain M, Funabashi M, Wong A, Pagé I (2019). Prevalence and practice characteristics of urban and rural or remote Australian chiropractors: analysis of a nationally representative sample of 1830 chiropractors. Aust J Rural Health.

[29] Bent A (1999). Allied health in Central Australia: Challenges and rewards in remote area practice. Aust J Physiother.

[30] Boshoff K, Hartshorne S (2008). Profile of occupational therapy practice in rural and remote South Australia. Aust J Rural Health.

[31] Merritt J, Perkins D, Boreland F (2013). Regional and remote occupational therapy: a preliminary exploration of private occupational therapy practice. Aust Occup Ther J.

[32] United Nations (2015). Sustainable development goals.

[33] Sobey AJ, Townsend NC, Metcalf CD, Bruce KD, Fazi FM (2013). Incorporation of Early Career Researchers within multidisciplinary research at academic institutions. Res Eval.

[34] Snowball JD, Shackleton CM (2018). Factors enabling and constraining research in a small, research-intensive South African University. Res Eval.

[35] Walters L, McGrail M, Carson D, O'Sullivan B, Russell D, Strasser R (2017). Where to next for rural general practice policy and research in Australia?. Med J Aust.

[36] Panaretto KS, Wenitong M, Button S, Ring IT (2014). Aboriginal community controlled health services: leading the way in primary care. Med J Aust.

[37] Greenhill J, Walker J, Playford D (2015). Outcomes of Australian rural clinical schools: a decade of success building the rural medical workforce through the education and training continuum. Rural Remote Health.

[38] Humphreys JS, Lyle D (2018). University Departments of Rural Health: is a national network of multidisciplinary academic departments in Australia making a difference?. Rural Remote Health.

[39] Australian Government Department of Health. Evaluation of the Rural Health Multidisciplinary Training (RHMT) Program 2019-2020. Canberra; 2020. https://www1.health.gov.au/internet/main/publishing.nsf/Content/rural-health-rhmt-evaluation. Accessed 27 Mar 2020.

[40] Bosanquet A, Mailey A, Matthews KE, Lodge JM (2017). Redefining ‘early career’ in academia: a collective narrative approach. Higher Educ Res.

[41] Denzin NK, Lincoln YS (2000). The handbook of qualitative research.

[42] Australian Government Department of Health (2016). The Modified Monash Model.

[43] Braun V, Clark V (2006). Using thematic analysis in psychology. Qual Res Psychol.

[44] McGrail M, O'Sullivan B, Russell D. Rural training pathways: the return rate of doctors to work in the same region as their basic medical training. Hum Resour Health. 2018;16:56. 10.1186/s12960-018-0323-7 PMC6198494 30348164

[45] McGrail M, O'Sullivan B, Russell D, Scott A. Solving Australia’s rural medical workforce shortage. 2017. http://mabel.org.au/__data/assets/pdf_file/0010/2294578/MABEL-policy-brief-no-3.pdf. Accessed 6 Aug 2020.

[46] Queensland Government (2007). Recognised rural generalist medicine.

[47] Rourke J. WHO Recommendations to improve retention of rural and remote health workers - important for all countries. Rural Remote Health. 2010;10:1654. . 21105750

[48] Cosgrave C, Malatzky C, Gillespie J (2019). Social determinants of rural health workforce retention: a scoping review. Int J Environ Res Public Health.

[49] Cosgrave C, Maple M, Hussain R (2018). An explanation of turnover intention among early-career nursing and allied health professionals working in rural and remote Australia - findings from a grounded theory study. Rural Remote Health.

[50] Strauss AL, JM C. (1998). Basics of qualitative research: techniques and procedures for developing grounded theory.

[51] Braun V, Clark V, Hayfield N, Terry G, Liamputtong P (2019). Thematic analysis. Handbook of research methods in health social sciences.

[52] Liamputtong P. Research methods in health: foundations for evidence-based practice. South Melbourne: Oxford University Press; 2013.

[53] Watt D (2007). On becoming a qualitative researcher: the value of reflexivity. Qual Rep.

[54] Workman B (2007). “Casing the joint”: explorations by the insider-researcher preparing for work-based projects. J Work Learn.

[55] Tracy SJ. Qualitative quality: eight 'big-tent' criteria for excellent qualitative research. Qualitative Inquiry. 2010;16:837–51.

[56] Fors M (2018). Geographical narcissism in psychotherapy: countermapping urban assumptions about power, space, and time. Psychoanal Psychol.

[57] Kerr J, Slocum W (2005). Managing corporate culture through reward systems. Acad Manage Exec.

[58] Durey A (2004). ‘Heroes and fairy wrens’: the changing face of rural general practice. Health Sociol Rev.

[59] Pyrko I, Dorfler V, Eden C (2017). Thinking together: what makes communities of practice work?. Hum Relat.

[60] Barclay L, Phillips J, Lyle D (2018). Rural and remote health research: does the investment match the need?. Aust J Rural Health.

[61] Australian Government Department of Health (2020). Medical research future fund strategies and priorities.

[62] Puljak L, Sharif WD (2009). Postdocs’ perceptions of work environment and career prospects at a US academic institution. Res Eval.

[63] Kulig JC (2010). Rural health research in Canada: assessing our progress. Can J Nurs Res.

